# Foveal Hypoplasia Related to Congenital Rubella

**DOI:** 10.7759/cureus.31766

**Published:** 2022-11-21

**Authors:** Ana Rita Viana, Rita Basto, Renato Correia Barbosa, Alexandre Silva, Carla Teixeira

**Affiliations:** 1 Department of Ophthalmology, Hospital Pedro Hispano, Unidade Local de Saúde de Matosinhos, Matosinhos, PRT

**Keywords:** ocular manifestations, foveal hypoplasia, rubella retinopathy, pigmentary retinopathy, congenital rubella syndrome

## Abstract

Normal development of the fovea begins before midgestation and continues for several years after birth. Foveal hypoplasia is a condition in which the foveal pit and the foveal avascular zone do not fully develop. Several diseases are known to be associated with foveal hypoplasia; however, a direct association between foveal hypoplasia and congenital rubella has not been stated so far. This report describes a case of foveal hypoplasia detected during adulthood in a patient with known fetal exposure to maternal rubella infection and several ocular features of congenital rubella syndrome, including microphthalmia, congenital cataract, and pigmentary retinopathy. During follow-up, the visual acuity and ocular fundus changes remained stable.

## Introduction

Normal development of the fovea begins in utero at fetal week 12. Around fetal week 25, the foveal pit begins its development with the centrifugal displacement of the inner retinal layers. The central-ward migration of cones and cone elongation are two later events that continue for several years after birth [1-3]. Foveal hypoplasia is a condition in which the foveal pit and the foveal avascular zone do not fully develop, and it has been associated with poor vision and nystagmus [4,5]. Several diseases are known to be associated with foveal hypoplasia, including albinism, *PAX6 *mutations and aniridia, *SLC38A8* mutations and related anterior segment dysgenesis, retinopathy of prematurity, optic nerve hypoplasia, achromatopsia, Stickler syndrome, familial exudative vitreoretinopathy, and incontinentia pigmenti [1,4]. There are also cases of isolated or idiopathic foveal hypoplasia [5].

To our knowledge, a direct association between foveal hypoplasia and congenital rubella has not been stated so far. The ocular pathologic features, most commonly observed in congenital rubella syndrome (CRS), are congenital cataracts, microphthalmia, and pigmentary retinopathy with a distinctive *salt-and-pepper* appearance [6]. Other common ocular features of CRS include congenital glaucoma, iris hypoplasia, nystagmus, concomitant strabismus, primary optic atrophy, and dacryostenosis [7].

This study aims to describe a case of foveal hypoplasia detected during adulthood associated with fetal exposure to maternal rubella during pregnancy. The diagnosis and clinical approach of the present clinical case took place in the Department of Ophthalmology of Hospital Pedro Hispano, Unidade Local de Saúde de Matosinhos, Portugal. This study was previously presented as a free paper at the 22nd Euretina Congress on September 2, 2022.

## Case presentation

A 52-year-old female with reported prenatal history of maternal rubella infection during pregnancy presented for ophthalmologic evaluation due to chronic bilateral visual impairment. She had undergone cataract surgery in her right eye at the age of 18 years. She was born full-term. She had no other relevant medical history. There was no family history of vision impairment. She denied nyctalopia.

**Figure 1:**
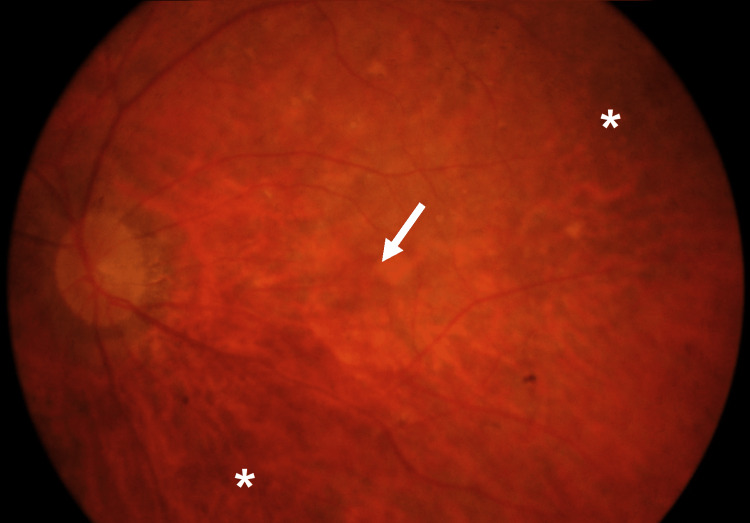
Ocular fundus of the left eye showing mild granular retinal pigment epithelium changes (asterisk), disorganized retinal vasculature, and lack of foveolar reflex (arrow).

Corrected distance visual acuity was *hand motion* in the right eye and 20/63 in the left eye. The right eye was microphthalmic and aphakic and presented torsional oscillatory nystagmus; the left eye presented no alterations apart from a mild cortical cataract. Both eyes presented a normal, dark iris and no signs of anterior segment dysgenesis. The intraocular pressure was normal in both eyes. Ocular fundus examination revealed mild diffuse granular retinal pigment epithelium changes, disorganized retinal vasculature, and lack of foveolar reflex in both eyes; the optic nerve head was normal (Figure 1).

**Figure 2:**
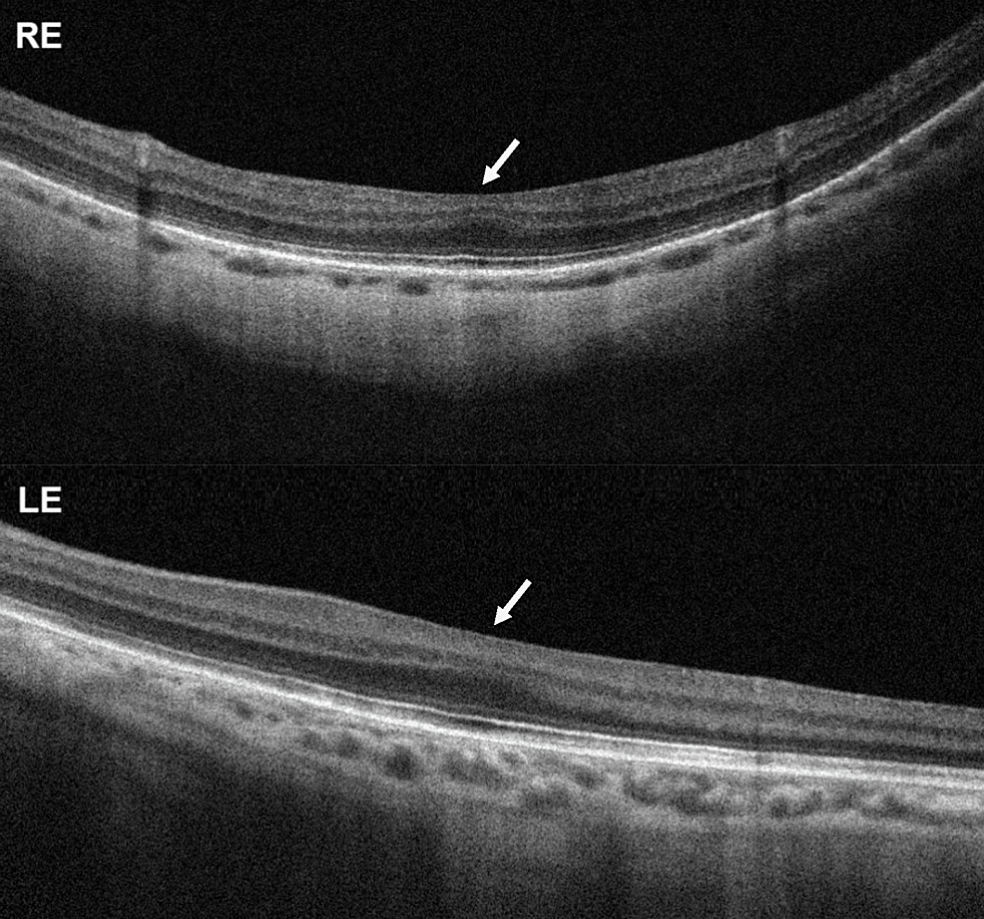
Spectral-domain optical coherence tomography horizontal transfoveolar scan showing bilateral foveal hypoplasia (arrow). RE, right eye; LE, left eye

**Figure 3:**
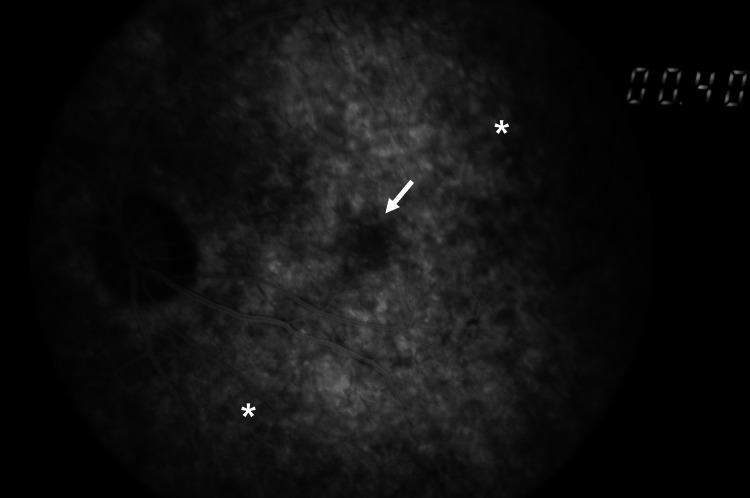
Fluorescein angiogram of the left eye demonstrating the absence of the foveal avascular zone (arrow) and diffuse mottling of the retinal pigment epithelium (asterisk).

Spectral-domain optical coherence tomography showed grade 3 bilateral foveal hypoplasia [1], with preserved outer nuclear layer widening but with the absence of the foveal pit, plexiform layer extrusion, and outer segments lengthening (Figure 2). Fluorescein angiography revealed the absence of the foveal avascular zone and a *salt-and-pepper* fundus appearance, with diffuse mottling of the retinal pigment epithelium, more evident in the periphery (Figure 3). Electroretinography showed normal retinal function in both eyes.

A systemic investigation was performed. She was seropositive for rubella-specific IgG antibodies. An echocardiogram revealed mild-to-moderate mitral insufficiency. There was no available genetic test.

The clinical condition was explained to the patient, and she maintained vigilance with regular ophthalmology examinations. During follow-up (>12 months), the visual acuity and ocular fundus changes remained stable.

## Discussion

CRS results from fetal exposure to maternal rubella during the first trimester [6]. The classic triad includes cataracts, congenital heart defects, and sensorineural deafness [8]. After infecting the placenta, the virus spreads through the fetal vascular system. Damage in the developing fetal organs can result from necrosis in the epithelium of chorionic villi, apoptosis of the infected cells due to direct viral damage, viral inhibition of mitosis and development of precursor cells, and ischemia secondary to the damage of endothelial cells of blood vessels [9].

In addition to pigmentary retinopathy and other pathologic ocular features most commonly observed in CRS, foveal alterations have also been described in patients with congenital rubella. Children with congenital rubella presented reduced central foveal thickness, subfoveal outer retinal thickness, and subfoveal choroid thickness in comparison with healthy children [6]. Morphologic features of the fovea in eyes with rubella retinopathy were also significantly different from healthy eyes, including a more U-shaped foveal dip [6]. Loss of the macular ring and foveolar reflexes were also described in children with rubella retinopathy [10]. In a case of suspected rubella retinopathy, optical coherence tomography imaging revealed foveal hypoplasia in both eyes; although central total retinal thickness was within the normal range, the outer nuclear layer thickness was slightly below the normal range [8].

Our patient presented disorganized retinal vasculature and foveal hypoplasia, in addition to several ocular pathologic features of CRS, including congenital cataracts, microphthalmia, and pigmentary retinopathy. Systemic and ocular signs of other known causes of foveal hypoplasia were not present, although a genetic test could not be performed to reliably exclude all possible genetic pathologies. Taking into account the clinical context, congenital rubella was the most likely etiology, and it is consistent with the existing literature [6,8,10]. As normal development of the fovea begins before midgestation [1-3], it may be compromised directly or indirectly by the rubella virus if the infection occurs in the first trimester. However, more investigations and case series are necessary to corroborate these findings.

## Conclusions

Rubella infection during the first trimester of pregnancy can have devastating consequences on fetal development. The eye is one of the organs frequently affected.

To date, a direct association between congenital rubella and foveal hypoplasia has not been defined. This case report suggests that foveal hypoplasia can also be found in patients with rubella retinopathy and that the critical early stages of foveal development can be compromised by the rubella virus. More studies are necessary to confirm these findings.
